# Inertial-Navigation-Aided Single-Satellite Highly Dynamic Positioning Algorithm

**DOI:** 10.3390/s19194196

**Published:** 2019-09-27

**Authors:** Lingling Zhang, Chengkai Tang, Yi Zhang, Houbing Song

**Affiliations:** 1 School of Marine Science and Technology, Northwestern Ploytechnical University, Xi’an 710072, China; llzhang@nwpu.edu.cn; 2 Shaanxi Key Laboratory of Integrated and Intelligent Navigation, Xi’an 710072, China; 3 School of Electronics and Information, Northwestern Polytechnical University, Xi’an 710072, China; 4 Department of Electrical, Computer, Software, and Systems Engineering, Embry-Riddle Aeronautical University, Daytona Beach, FL 32114, USA; Houbing.Song@erau.edu

**Keywords:** single-satellite system, highly dynamic positioning, inertial navigation system (INS), unscented Kalman Filter (UKF), pseudorange difference

## Abstract

Nowadays, research on global navigation satellite systems (GNSS) has reached a certain level of maturity to provide high-precision positioning services in many applications. Nonetheless, there are challenging GNSS-denial environments where a temporarily deployed single-satellite positioning system is a promising choice. To further meet the emergency call of highly dynamic targets in such situations, an augmented single-satellite positioning algorithm is proposed in this paper. First, the initial location of the highly dynamic target is found by real-time displacement feedback from the inertial navigation system (INS). Then, considering the continuity of position change, and taking advantage of the high accuracy and robustness of the unscented Kalman filter (UKF), target location is through iteration and fusion. Comparing this proposed method with the least-squares Newton-iterative Doppler single-satellite positioning system and the pseudorange rate-assisted method under synthetic error conditions, the positioning error of our algorithm was 10% less than the other two algorithms. This verified the validation of our algorithm in the single-satellite system with highly dynamic targets.

## 1. Introduction

Today’s global navigation satellite systems (GNSS) have excellent performance and high positioning accuracy in most scenarios. With the support of GNSS constellation, they can satisfy the daily positioning requirements of military and civilian navigation. However, the inherent vulnerability of constellation GNSSs significantly degrade their positioning ability or even make them completely unable to locate under extreme conditions, such as during wartime. To obtain locations precisely in emergency environments, a rapidly deployed navigation and positioning system is imperative. Single-satellite systems show apparent advantages with fast deployment and low cost compared to GNSSs. Thus, it is an essential supplement to GNSSs. Existing single-satellite positioning techniques adopt observation values at different times from the only satellite to locate. The literature [1] proposed a frequency-measurement-based single-satellite positioning method that utilized the frequency difference of arrival during satellite movement. However, this method has low positioning accuracy and is limited to stationary or small dynamic targets. The literature [2] found the geometric relationship between location and rate of phase difference, Doppler frequency and satellite ephemeris position to obtain positioning results but it is quite sensitive to the parameters. The literature [3] also proposed an angular-measurement-based single-satellite positioning method that utilized the angular-velocity relationship between satellite and target to achieve positioning. However, it requires high accuracy of satellite ephemeris. The literature [4,5] proposed a Doppler-based single-satellite positioning method that utilized frequency difference to construct the positioning hyperboloid and integrated it with the inertial navigation system (INS) to achieve positioning. However, these methods are more suitable for static positioning, with low positioning accuracy and fast divergence for dynamic targets. The literature [6] proposed radial acceleration measurement based on a single-satellite positioning method. This method is simple since it only requires radial acceleration but, as the data source is limited, its reliability is poor. The literature [7] proposed a beam-scanning single-satellite positioning method with high positioning accuracy for static targets. However, it works in active location mode, which needs interaction with the data center. The literature [8] also proposed a BP neural-network-based single-satellite positioning method. The positioning results were stable but it takes a lot of time to perform the learning and updating process, so practical applications are limited. Researcher [9] also proposed an INS-based Doppler integration single-satellite positioning method that has a good positioning effect for both dynamic and static targets. However, since the final filtering result depends on the least-squares solution result, the stability of this method is limited. In the above algorithms, the main method is to transplant the GPS positioning method directly to the single-satellite positioning system. The problem of keeping the target immovable in single-satellite positioning systems is neglected. To answer this question, an inertial-navigation-aided single-satellite highly dynamic positioning algorithm is proposed. First, we computed and compensated the displacement of the moving target by INS [10]. Then, we utilized the integral Doppler pseudorange to construct an equal-frequency cone surface and use the height information of the moving target to establish the height surface. Next, we obtained the coarse positioning solution with these surfaces. Finally, the displacement value provided by INS was calculated and corrected in the UKF filter to improve the single-satellite positioning result.

## 2. Single-Satellite System Model

**Figure 1:**
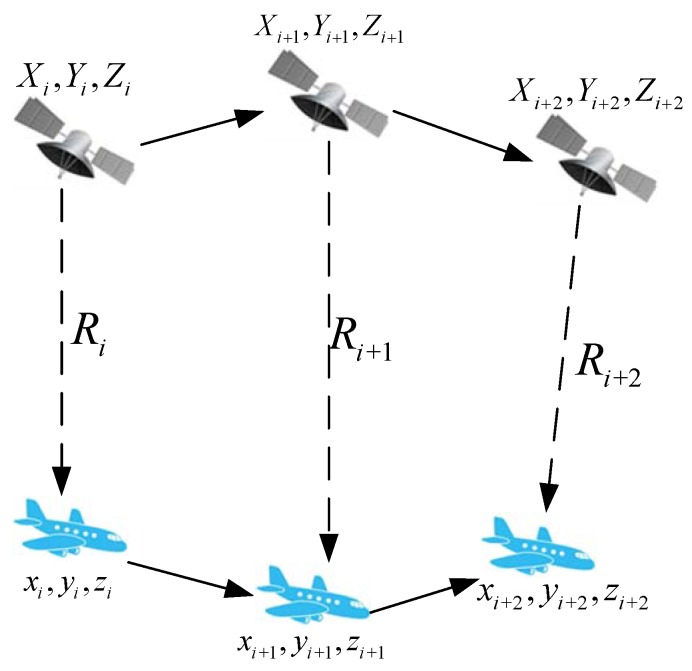
Single-satellite positioning based on inertial navigation system (INS).

Under the conditions of GNSS denial, a single-satellite positioning system could be an effective complement [11]. Most targets, such as vehicles, personnel, ships and aircraft are dynamic, that is to say, their track, direction and movement time are uncertain. Therefore, in this paper, inertial navigation is the motion of the target that must be compensated by the INS. The principle of the inertial-navigation-aided single-satellite positioning method is shown in Figure 1.

Here, $\left\{X_{i},Y_{i},Z_{i}\right\}$ denotes the satellite position at time *i*, $R_{i}$ denotes the pesudorange between satellite and target at time *i* and $\left\{x_{i},y_{i},z_{i}\right\}$ denotes the position of the target at time *i*. Then, the pesudorange at the three successive moments, as shown in Figure 1, can be described as

(1) $$R_{i}=\sqrt{{\left(X_{i}-x_{i}\right)}^{2}+{\left(Y_{i}-y_{i}\right)}^{2}+{\left(Z_{i}-z_{i}\right)}^{2}}+\delta t_{u}$$

(2) $$R_{i+1}=\sqrt{{\left(X_{i+1}-x_{i+1}\right)}^{2}+{\left(Y_{i+1}-y_{i+1}\right)}^{2}+{\left(Z_{i+1}-z_{i+1}\right)}^{2}}+\delta t_{u}$$

(3) $$R_{i+2}=\sqrt{{\left(X_{i+2}-x_{i+2}\right)}^{2}+{\left(Y_{i+2}-y_{i+2}\right)}^{2}+{\left(Z_{i+2}-z_{i+2}\right)}^{2}}+\delta t_{u}$$

where $\delta t_{u}$ represents the error caused by the receiver-clock offset.

With the aid of inertial navigation [12], we can obtain the position of the target at these three successive moments as

(4) $$\begin{array}{ccc} x_{i+1} & = & x_{i}+\Delta x_{i}\\ y_{i+1} & = & y_{i}+\Delta y_{i}\\ z_{i+1} & = & z_{i}+\Delta z_{i} \end{array}$$

(5) $$\begin{array}{ccc} x_{i+2} & = & x_{i+1}+\Delta x_{i+1}\\ y_{i+2} & = & y_{i+1}+\Delta y_{i+1}\\ z_{i+2} & = & z_{i+1}+\Delta z_{i+1}, \end{array}$$

where $\Delta x_{i},\Delta y_{i},\Delta z_{i}$ represent the displacement of the target by the INS from the *i*th time point to the i+1th time point, respectively. we substituted (4) and (5) into Equations (2) and (4) and the pseudorange between the dynamic targets and the satellites is given in simplified form:

(6) $$\begin{array}{ccc} R_{i} & = & \sqrt{{\left(R_{i,x}\right)}^{2}+{\left(R_{i,y}\right)}^{2}+{\left(R_{i,z}\right)}^{2}}+\delta t_{u}\\ R_{i,x} & = & X_{i+1}-\left(x_{i}+\Delta x_{i}\right)\\ R_{i,y} & = & Y_{i+1}-\left(y_{i}+\Delta y_{i}\right)\\ R_{i,z} & = & Z_{i+1}-\left(z_{i}+\Delta z_{i}\right) \end{array}$$

(7) $$\begin{array}{ccc} R_{i+2} & = & \sqrt{{\left(R_{i+2,x}\right)}^{2}+{\left(R_{i+2,y}\right)}^{2}+{\left(R_{i+2,z}\right)}^{2}}+\delta t_{u}\\ R_{i+2,x} & = & X_{i+2}-\left(x_{i}+\Delta x_{i}+\Delta x_{i+1}\right)\\ R_{i+2,y} & = & Y_{i+2}-\left(y_{i}+\Delta y_{i}+\Delta y_{i+1}\right)\\ R_{i+2,z} & = & Z_{i+2}-\left(z_{i}+\Delta z_{i}+\Delta z_{i+1}\right) \end{array}$$

There are four unknowns in the three above equations, so we could not settle the position [13]. In addition, we included the height measurement that was directly obtained from the altimeter at time *i* to determine the target position, since it satisfies

(8) $$h_{i}=\sqrt{x_{i}^{2}+y_{i}^{2}+z_{i}^{2}}$$

The initial estimation of the target position at time point *i*th can be obtained by a least-mean-square algorithm. Based on the Taylor expansion, the estimation problem can be described as

(9) G·x=b

where

(10) $$G={\left[G_{1},G_{2},G_{3},G_{4}\right]}^{T}$$

(11) $$x=\left[\begin{array}{c} x_{i}\\ y_{i}\\ z_{i} \end{array}\right]$$

and

(12) $$G_{m}=\left[\begin{array}{c} -\frac{x_{i+m,k-1}}{R_{i+m,k-1}}\\ -\frac{y_{i+m,k-1}}{R_{i+m,k-1}}\\ -\frac{z_{i+m,k-1}}{R_{i+m,k-1}}\\ 1 \end{array}\right]\;,m=0,1,2$$

(13) $$R_{i+m,k-1}=\sqrt{{\left(X_{i+m}-x_{i+m,k-1}\right)}^{2}+{\left(Y_{i+m}-y_{i+m,k-1}\right)}^{2}+{\left(Z_{i+m}-z_{i+m,k-1}\right)}^{2}}$$

(14) $$G_{4}=\left[\begin{array}{c} -\frac{x_{i+1,k-1}}{\sqrt{x_{i+1,k-1}^{2}+y_{i+1,k-1}^{2}+z_{i+1,k-1}^{2}}}\\ -\frac{y_{i+1,k-1}}{\sqrt{x_{i+1,k-1}^{2}+y_{i+1,k-1}^{2}+z_{i+1,k-1}^{2}}}\\ -\frac{z_{i+1,k-1}}{\sqrt{x_{i+1,k-1}^{2}+y_{i+1,k-1}^{2}+z_{i+1,k-1}^{2}}}\\ 0 \end{array}\right]$$

(15) $$b=\left[\begin{array}{c} R_{i}-R_{i,k-1}-\delta t_{u,k-1}\\ R_{i+1}-R_{i+1,k-1}-\delta t_{u,k-1}\\ R_{i+2}-R_{i+1,k-1}-\delta t_{u,k-1}\\ h_{i+1}-\sqrt{x_{i+1,k-1}^{2}+y_{i+1,k-1}^{2}+z_{i+1,k-1}^{2}} \end{array}\right]$$

Equation (10) contains four unknown parameters. The least-squares method was utilized to jointly solve the dynamic results at each iteration [14], Δx, as follows:

(16) $$\Delta x={\left(G^{T}G\right)}^{-1}G^{T}b$$

(17) $$\left[\begin{array}{c} x_{i+1,k}\\ y_{i+1,k}\\ z_{i+1,k} \end{array}\right]=\left[\begin{array}{c} x_{i+1,k-1}\\ y_{i+1,k-1}\\ z_{i+1,k-1} \end{array}\right]+\left[I_{3},0_{3\times 1}\right]\Delta x$$

By repeating iterations until increment ∥Δx∥ met a certain accuracy threshold, we could obtain the initial estimation of the target’s three-dimensional coordinates.

## 3. Navigation-Data Fusion

Considering the complex and changeable motion of the target and the inherent error of the INS, the initial estimation of the target position from the previous section needs to further be improved [15]. The traditional Kalman filter is based on the linear model, shows high positioning fluctuations and suffers divergence in sudden changes. Therefore, in this section, we present an unscented Kalman filter (UKF) -based inertial-navigation-aided single-satellite highly dynamic positioning algorithm.

Suppose x is a *l* dimensional random variable that follows Gaussian distribution, that is, $x\;∼\;N(\bar{x},P)$, where $\bar{x}$ is an *l* dimensional vector and P is a l×l symmetric semipositive definite square matrix. P is decomposed by Cholesky decomposition as $P=\dot{P}\cdot {\dot{P}}^{T}$, where $\dot{P}$ is the lower triangular matrix. A new posterior random variable y=f(x) is obtained through nonlinear transformation.

In this case, unscented transformation (UT) is utilized to estimate the mean value and the covariance matrix of y. Choosing a set of 2l+1 sampling points that meet the selection scheme as

(18) $$\begin{array}{ccc} \xi_{0} & = & \bar{x}\\ \xi_{i} & = & \bar{x}+r{\dot{P}}_{i}\\ \xi_{i+1} & = & \bar{x}-r{\dot{P}}_{i}\\ i & = & 1,3,\cdots ,2l-1 \end{array}$$

where $r=\sqrt{l+\gamma }$ and $\gamma =a^{2}\left(l+\kappa \right)-l$ represent the main scale parameter and κ represents a minor scale parameter; *a* indicates the extent to which the sampling point deviates from the expected value, which is usually a very small positive number. It can be seen that all $\xi_{i}$ and $\xi_{i+1}$ are symmetrical about $\xi_{0}=\bar{x}$, so weight factors can be defined as

(19) $$\begin{array}{ccc} w_{0} & = & \frac{\gamma }{l+\gamma }\\ v_{0} & = & \frac{\gamma }{l+\gamma }+1-a^{2}\\ w_{i} & = & v_{i}=\frac{1}{2(l+\gamma )}\\ i & = & 1,2,\cdots ,2l \end{array}$$

The predicted state mean and covariance matrix can be obtained through the weighted sum.

(20) $$\bar{x}=\sum_{i=0}^{2l}w_{i}\xi_{i}$$

(21) $$P=\sum_{i=0}^{2l}v_{i}\left(\xi_{i}-\bar{x}\right){\left(\xi_{i}-\bar{x}\right)}^{T}$$

Propagating each sampling point through nonlinear function,

(22) $$\begin{array}{ccc} \eta_{0} & = & f\left(\xi_{0}\right)=f\left(\bar{x}\right)\\ \eta_{i} & = & f\left(\xi_{i}\right)=f\left(\bar{x}+\sqrt{l+\gamma }{\dot{P}}_{i}\right)\\ \eta_{i+1} & = & f\left(\xi_{i+1}\right)=f\left(\bar{x}-\sqrt{l+\gamma }{\dot{P}}_{i}\right)\\ i & = & 1,3,\cdots ,2l-1 \end{array}$$

Then, the predicted measurement mean and covariance matrix can be obtained through the weighted sum.

(23) $$\bar{y}=\Theta \left(\eta \right)=\sum_{i=0}^{2l}w_{i}\eta_{i}$$

(24) $$P_{y}=\Psi \left(\eta ,\bar{y}\right)=\sum_{i=0}^{2l}v_{i}\left(\eta_{i}-\bar{y}\right){\left(\eta_{i}-\bar{y}\right)}^{T}$$

The UKF replaced the traditional EKF to solve nonlinear problems through unscented transformation. This transformation approximates the probability of distribution rather than the nonlinear function and avoids calculation of the Jacobi matrix. It provides estimation accuracy approximately to the third-order EKF, equally computationally intensive with the first-order EKF. In a single-satellite system, the decremental of computation load has great significance for performance improvement in highly dynamic positioning.

In the inertial-navigation-aided single-satellite system, the augmented state is denoted as

(25) $$X_{t}={\left[\begin{array}{ccccccccc} x & y & z & v_{x} & v_{y} & v_{z} & a_{x} & a_{y} & a_{z} \end{array}\right]}_{t}$$

where ${\left[x,y,z\right]}_{t}$, ${\left[v_{x},v_{y},v_{z}\right]}_{t}$ and ${\left[a_{x},a_{y},a_{z}\right]}_{t}$ represent the position, velocity and acceleration of the positioning target at time *t*, respectively. The state-space equation model to describe the dynamic process of targets:

(26) $${\bar{X}}_{t+1}=FX_{t}+W_{t}$$

where, F denotes a dynamic matrix, dynamic matrix $F_{1}$ represents speed variation and $F_{2}$ represents acceleration variation; $W_{t}$ denotes single-satellite-system noise at time t. In practice, the obtained samples are discrete values. The state-transition matrix obtained by discretizing F can be regarded as nontime-varying when the sampling interval is small.

(27) $$F_{1}=\left[\begin{array}{ccccccccc} 0 & 0 & 0 & 1 & 0 & 0 & 0 & 0 & 0\\ 0 & 0 & 0 & 0 & 1 & 0 & 0 & 0 & 0\\ 0 & 0 & 0 & 0 & 0 & 1 & 0 & 0 & 0\\ 0 & 0 & 0 & 0 & 0 & 0 & 1 & 0 & 0\\ 0 & 0 & 0 & 0 & 0 & 0 & 0 & 1 & 0\\ 0 & 0 & 0 & 0 & 0 & 0 & 0 & 0 & 1\\ 0 & 0 & 0 & 0 & 0 & 0 & 0 & 0 & 0\\ 0 & 0 & 0 & 0 & 0 & 0 & 0 & 0 & 0\\ 0 & 0 & 0 & 0 & 0 & 0 & 0 & 0 & 0 \end{array}\right]$$

(28) $$F_{2}=\left[\begin{array}{ccccccccc} 0 & 0 & 0 & 0 & 0 & 0 & 1 & 0 & 0\\ 0 & 0 & 0 & 0 & 0 & 0 & 0 & 1 & 0\\ 0 & 0 & 0 & 0 & 0 & 0 & 0 & 0 & 1\\ 0 & 0 & 0 & 0 & 0 & 0 & 0 & 0 & 0\\ 0 & 0 & 0 & 0 & 0 & 0 & 0 & 0 & 0\\ 0 & 0 & 0 & 0 & 0 & 0 & 0 & 0 & 0\\ 0 & 0 & 0 & 0 & 0 & 0 & 0 & 0 & 0\\ 0 & 0 & 0 & 0 & 0 & 0 & 0 & 0 & 0\\ 0 & 0 & 0 & 0 & 0 & 0 & 0 & 0 & 0 \end{array}\right]$$

The prediction and update steps of UKF are looped after initialization. Assuming that the state of a single-satellite system is a random variable $X_{t}\in R^{n}$, with expected value ${\bar{X}}_{t}$ and covariance matrix $P_{t}$, by supplying that UKF a prior estimate ${\bar{X}}_{0}$ and covariance $P_{0}$, the next step is to build a state-transition matrix $X_{t}=f\left(X_{t-1},w_{t-1},u_{t-1}\right)$. Let Δt represents the sampling interval and φ represents the following state-transition matrix.

(29) $$\varphi =\left[\begin{array}{ccccccccc} 1 & 0 & 0 & \Delta t & 0 & 0 & \frac{\Delta t^{2}}{2} & 0 & 0\\ 0 & 1 & 0 & 0 & \Delta t & 0 & 0 & \frac{\Delta t^{2}}{2} & 0\\ 0 & 0 & 1 & 0 & 0 & \Delta t & 0 & 0 & \frac{\Delta t^{2}}{2}\\ 0 & 0 & 0 & 1 & 0 & 0 & \Delta t & 0 & 0\\ 0 & 0 & 0 & 0 & 1 & 0 & 0 & \Delta t & 0\\ 0 & 0 & 0 & 0 & 0 & 1 & 0 & 0 & \Delta t\\ 0 & 0 & 0 & 0 & 0 & 0 & 1 & 0 & 0\\ 0 & 0 & 0 & 0 & 0 & 0 & 0 & 1 & 0\\ 0 & 0 & 0 & 0 & 0 & 0 & 0 & 0 & 1 \end{array}\right]$$

State quantity was utilized to construct the sampling points and calculate the unscented weight coefficients; ${\bar{X}}_{t}^{-}$ represents the predicted estimate at sampling interval *t* and ${\bar{X}}_{t}$ represents the posterior estimate at sampling interval *t*. In the time-update part of single-satellite positioning, the state transformation of the generated test point set is first carried out. Since UKF guarantees second-order accuracy, nonlinear transformation function f() can be rewritten as

(30) $$\xi_{t}^{-}=f\left(\xi_{t-1}\right)=\varphi \xi_{t-1}$$

The predicted estimate of $X_{t}$ is:

(31) $${\bar{X}}_{t}^{-}=\sum_{i=0}^{2l}w_{i}{\left(\xi_{t-1}\right)}_{i}$$

Construction of measurement equation vector $Z_{t}$ by using a pseudorange and height values at *t* time:

(32) $$Z_{t}=\left[\begin{array}{c} R_{t}\\ R_{t+1}\\ R_{t+2}\\ h_{t+1} \end{array}\right]$$

Similar to the least-squares part, the state variable substituting value $X_{t}$ is the target-state value at t+1 time; $\delta x_{i},\delta y_{i},\delta z_{i}$ are the relative displacements of the three-dimensional coordinates between time *i* and i+1, recorded by the inertial navigation system; $p_{i}^{x},p_{i}^{y},p_{i}^{z}$ represent the coordinates of the single satellite at time *i*; $R_{i}$ represents the actual pseudorange value between single satellite and target at time *i*; ${\hat{R}}_{i}$ represents the estimated pseudorange value at time *i*; $h_{i}$ represents the target-height value at time *i*; ${\hat{h}}_{i}$ represents the estimated target-height value at time *i*. Here, $\eta_{t}=h\left(\xi_{t-1}^{-}\right)$ was utilized to calculate the pseudorange value of $\xi_{t-1}^{-}$ at the corresponding time.

(33) $${\hat{\eta }}_{i,k}^{-}=\sqrt{{\left({\hat{\xi }}_{i+1,x,k}^{-}-\Delta x_{i}-p_{i}^{x}\right)}^{2}+{\left({\hat{\xi }}_{i+1,y,k}^{-}-\Delta y_{i}-p_{i}^{y}\right)}^{2}+{\left({\hat{\xi }}_{i+1,z,k}^{-}-\Delta z_{i}-p_{i}^{z}\right)}^{2}}$$

(34) $${\hat{\eta }}_{i+1,k}^{-}=\sqrt{{\left({\hat{\xi }}_{i+1,x,k}^{-}-p_{i+1}^{x}\right)}^{2}+{\left({\hat{\xi }}_{i+1,y,k}^{-}-p_{i+1}^{y}\right)}^{2}+{\left({\hat{\xi }}_{i+1,z,k}^{-}-p_{i+1}^{z}\right)}^{2}}$$

(35) $${\hat{\eta }}_{i+2,k}^{-}=\sqrt{{\left({\hat{\xi }}_{i+1,x,k}^{-}+\Delta x_{i+1}-p_{i+2}^{x}\right)}^{2}+{\left({\hat{\xi }}_{i+1,y,k}^{-}-\Delta y_{i+1}-p_{i+2}^{y}\right)}^{2}+{\left({\hat{\xi }}_{i+1,z,k}^{-}-\Delta z_{i+1}-p_{i+2}^{z}\right)}^{2}}$$

(36) $$\begin{array}{ccc} {\hat{\eta }}_{h,i+1,k}^{-} & = & \sqrt{{\left({\hat{\xi }}_{i+1,x,k}^{-}\right)}^{2}+{\left({\hat{\xi }}_{i+1,y,k}^{-}\right)}^{2}+{\left({\hat{\xi }}_{i+1,z,k}^{-}\right)}^{2}}\\ k & = & 1,2,\cdots ,2l+1 \end{array}$$

All symbols in Equations (36–39) are scalars. For example, ${\hat{\eta }}_{i+1,k}^{-}$ represents the *k*th column of the solution of vector ${\hat{\eta }}_{t}^{-}$ at time *i*; ${\hat{\xi }}_{i+1,x,k}^{-}$, ${\hat{\xi }}_{i+1,y,k}^{-}$ and ${\hat{\xi }}_{i+1,z,k}^{-}$ represent the x,y,z value of the *k*th column of solution of vector $\eta_{t}^{-}$ at time i+1. Then, predictive value ${\hat{z}}_{t}^{-}$ of $z_{t}$ is as follows:

(37) $${\hat{z}}_{t}^{-}=\sum_{i=0}^{2l}w_{i}{\left(\eta_{t}^{-}\right)}_{i}$$

UT transformation was utilized to obtain a priori covariance matrix $P_{t}^{-}$

(38) $$P_{t}^{-}=\sum_{i=0}^{2l}w_{i}\left(\xi_{t}^{-}-{\hat{X}}_{t}^{-}\right){\left(\xi_{t}^{-}-{\hat{X}}_{t}^{-}\right)}_{t}^{T}$$

In order to make the dynamic positioning prediction accurate, initialization was only performed once and the measurement-update part was as follows:

(39) $$P_{t}^{-}=\sum_{i=0}^{2l}w_{i}\left(\xi_{t}^{-}-{\hat{X}}_{t}^{-}\right){\left(\xi_{t}^{-}-{\hat{X}}_{t}^{-}\right)}_{i}^{T}$$

In order to make the dynamic positioning prediction accurate, initialization was only performed once and the measurement-update part was as follows:

(40) $$P_{t}^{yy}=\sum_{i=0}^{2l}w_{i}\left(\eta_{t}^{-}-{\hat{z}}_{t}^{-}\right){\left(\eta_{t}^{-}-{\hat{z}}_{t}^{-}\right)}_{i}^{T}$$

(41) $$P_{t}^{xy}=\sum_{i=0}^{2l}w_{i}\left(\xi_{t}^{-}-{\hat{X}}_{t}^{-}\right){\left(\eta_{t}^{-}-{\hat{z}}_{t}^{-}\right)}_{i}^{T}$$

(42) $${\hat{X}}_{t}={\hat{X}}_{t}^{-}+k_{t-1}\left(z_{t}-{\hat{z}}_{t}^{-}\right)$$

(43) $$k_{t}=P_{t}^{xy}{\left(P_{t}^{xy}\right)}^{-1}$$

(44) $$P_{t}=P_{t}^{-}-k_{t}P_{t}^{yy}k_{t}^{T}$$

The Kalman filter gives the estimated value at the new time point according to the a priori estimate. The $\hat{\cdot }$ of the state variable indicates the prediction estimate, right label-indicates the a priori value, $k_{t}$ indicates the Kalman filter coefficient matrix at time *t* and $P_{t}^{xy}$ and $P_{t}^{yy}$ are the prediction covariance error matrix of the single-satellite navigation system. The computational complexity of the UKF filter was smaller than that of the EKF with the same accuracy and tracking performance was also better than EKF. It is very suitable for dynamic-target location and has good robustness. Combining INS input compensation and the initial least-squares calculation value, the UKF filter can provide stable rough positioning for a considerable period of time and can meet the requirements of single-satellite navigation.

## 4. Simulation and Result Analysis

In the simulation part, low-orbit satellites were utilized with a distance of 1200 km from the ground near Earth’s surface.The advantage of low-orbit satellites are obviously Doppler integral changes, which are beneficial to improve the positioning accuracy of single-satellite navigation. The designed run period was 2.13 h; orbit inclination was π/6; right ascension point was π/3; the eccentricity of the elliptical orbit was 0.1; and perigee angle was pi/6.

The main sources of error for single-satellite positioning include orbital, pseudorange, satellite-velocity and INS errors. These are considered in single- and comprehensive-factor simulations. Due to INS errors existing in all target-displacement data, every single-factor simulation includes INS errors. At the same time, our proposed algorithm was compared witha Doppler single-satellite positioning method based on the least-square Newtonian iteration and EKF single-satellite Doppler pseudorange positioning methods.

### 4.1. Orbital-Error Analysis

**Figure 2:**
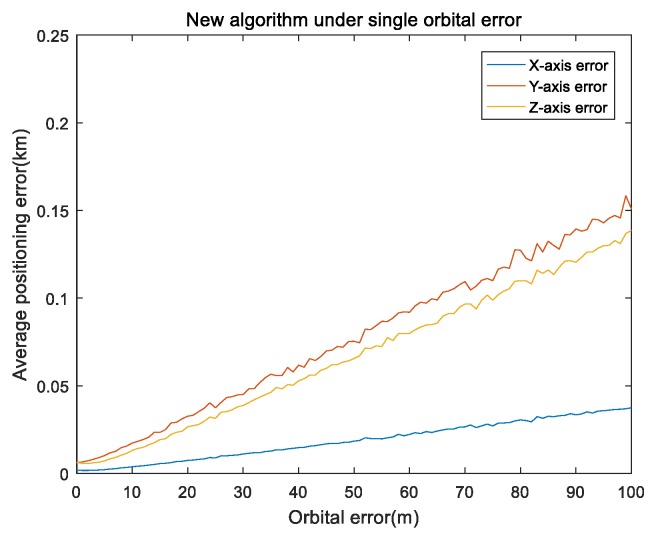
Our algorithm under a single orbital error of the x,y,z axis.

**Figure 3:**
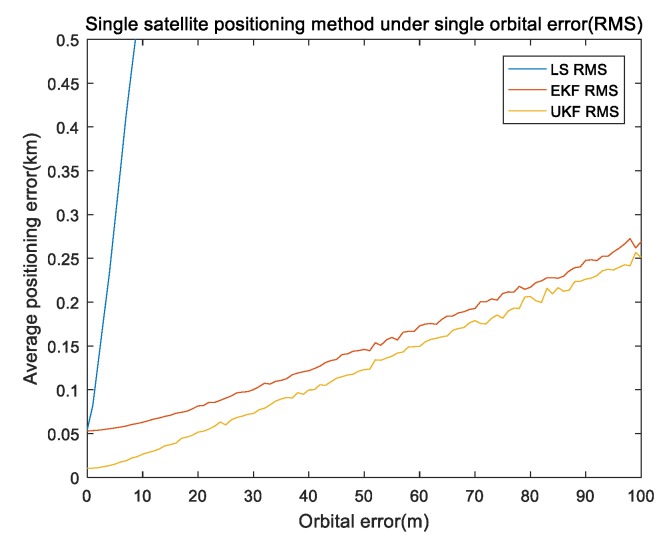
Performance comparison of different algorithms under a single orbital error.

In the first part, considering the impact of orbit-determination error on the single-satellite positioning results, we set a simulation time at 3000 s and single integration time at 150 s.The inertial component parameters that were utilized in the actual measurement of INS data included gyro zero bias $10^{-3\circ }/h$; gyro random walk was $10^{-4\circ }/sqrt\left(h\right)$; initial attitude error was $2^{\prime \prime },2^{\prime \prime },2^{\prime \prime }$; INS initial error was 20 m; movement speed was 20 m/s. The main direction of the target was the *y* axis direction and a height error of 1 m was included in the height direction. In order to eliminate random errors, the orbit-determination error was taken from 0 to 100 m at intervals of 1 m and the remaining errors were all 0. The simulation was repeated 100 times under the same experiment conditions. Figure 2 and Figure 3 show the results of the orbit error.

Figure 2 shows the mean of the positioning errors of the x,y,z axis for different orbital errors. It can be seen in Figure 2 that the positioning error was positively correlated with the orbital error; at the same time, the *y* and *z* axis errors were greater than that of the *x* axis because the movement direction was mainly on the *y* axis and height error was added in the *z* axis. Figure 3 shows the performance comparison of the absolute error values (referred to as RMS) for the three methods under single orbital-error conditions, where the error of the least-squares positioning algorithm was much larger than that of the other two algorithms. To facilitate comparison, the display range of the shaft was reduced. It can be seen in Figure 3 that our proposed algorithm was superior to the EKF algorithm, which is much better than the algorithm of least squares-Newton iteration.

### 4.2. Pseudorange Error Analysis

**Figure 4:**
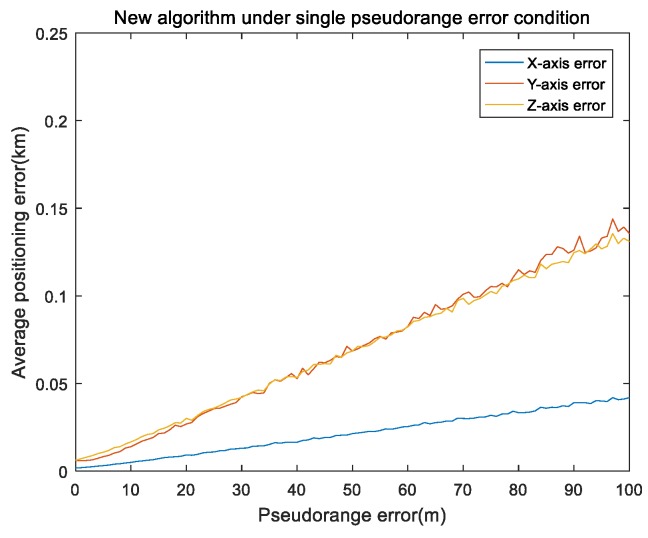
Our algorithm under single pseudorange error of the x,y,z axis.

**Figure 5:**
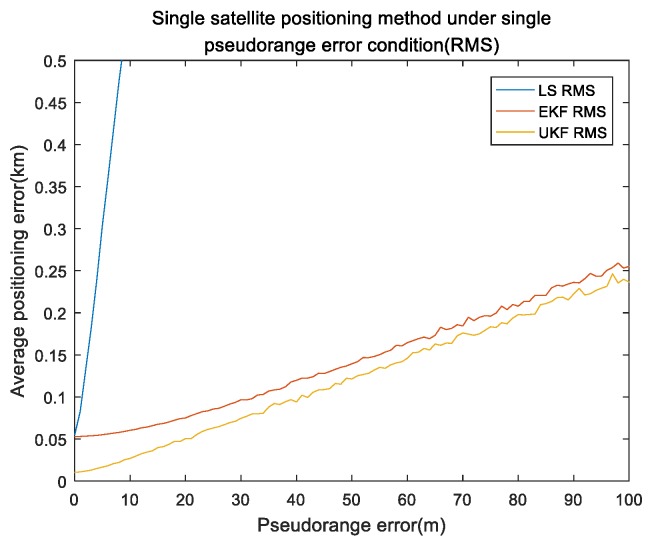
Performance comparison of different algorithms under single pseudorange error.

Pseudorange error is a main error source in single-satellite positioning. For the pseudorange error, simulation time was set to 3000 s and the single integration time was 150 s. The INS setting was the same as that in the orbit part. Pseudorange error was set from 0 to 100 m, with an interval of 1 m. The remaining errors were all 0. The simulation was repeated 100 times under the same experiment conditions. Figure 4 and Figure 5 show the results of the pseudorange error.

Figure 4 shows the mean of the positioning errors of the x,y,z axis for different pseudorange errors. It can be seen that the pseudorange error was positively correlated with the positioning error. Figure 5 shows the performance comparison of absolute error values (referred to as RMS) for the three methods under single pseudorange-error conditions, the RMS value of every algorithm was approximately linear with the pseudorange value. Our proposed algorithm was better than the EKF algorithm, and far better than the least-squares Newton iteration algorithm.

### 4.3. Satellite-Velocity-Error Analysis

**Figure 6:**
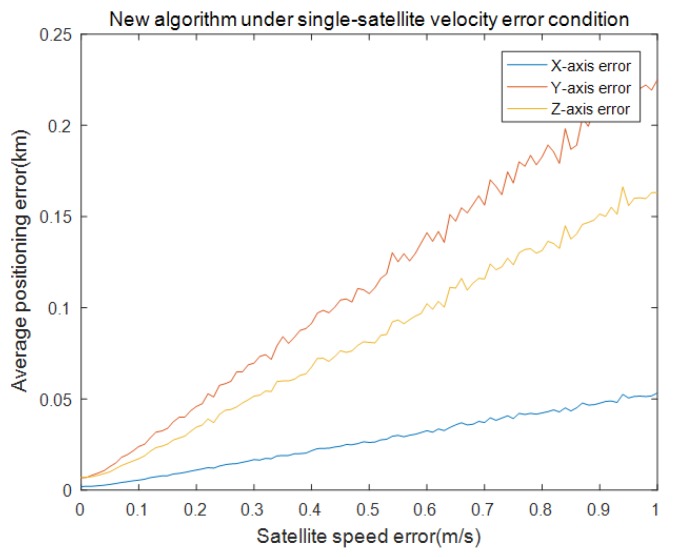
Our algorithm under single-satellite velocity error of x,y,z axis.

**Figure 7:**
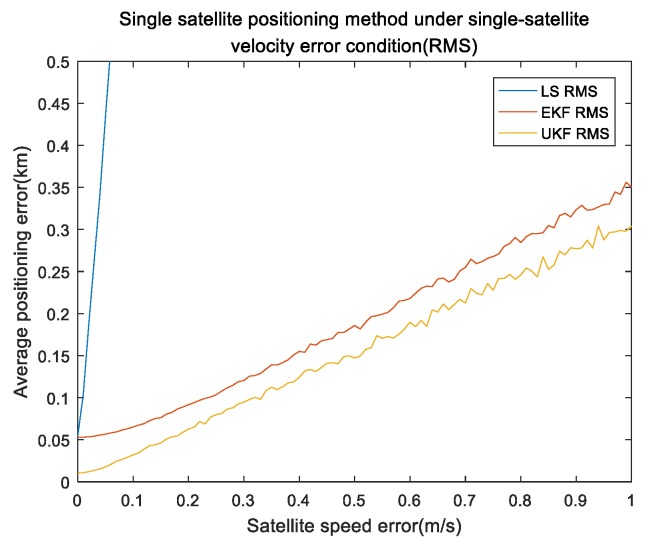
Performance comparison of different algorithms under single-satellite velocity error.

In this part, the influence of satellite-speed error is considered in single-satellite positioning. We set the simulation time to 3000 s and the single-integration time was 150 s. The INS setting was the same as the pseudorange case. Satellite-speed error was from 0 to 1 m/s and the interval was 0.02 m/s.The remaining errors were all 0. The simulation was repeated 100 times under the same experiment conditions. Figure 6 and Figure 7 show the results of the satellite-velocity error.

Figure 6 shows the mean of the positioning errors of the x,y,z axis for different satellite-velocity errors. It can be seen that the results were very close to the pseudorange results but the satellite-velocity error was 0.5 m/s, the position error of the three axes was 120,75,24 m and was larger than the pseudorange-error results. This means that the effect of the satellite-velocity error was larger than that of the pseudorange error in the single-satellite position. Figure 5 shows the performance comparison of the error absolute values (referred to as RMS) for the three methods under single-satellite-velocity error conditions. It can be seen that the RMS value of every algorithm was approximately linear to the satellite-velocity value and our proposed UKF algorithm was also superior to the other algorithms; the EKF algorithm was better than the least-squares Newton iteration algorithm.

### 4.4. Comprehensive Measured Environmental-Error Analysis

**Figure 8:**
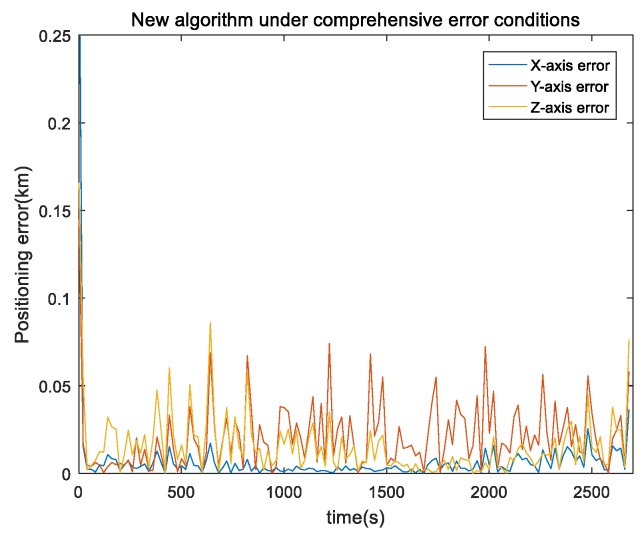
Our algorithm error of the x,y,z axis under a comprehensive environment.

**Figure 9:**
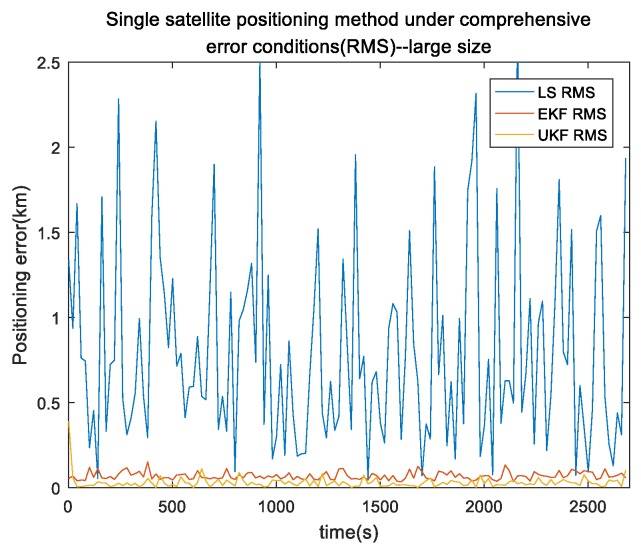
Performance comparison of different algorithms under a comprehensive environment.

In the actual single-satellite positioning system, all types of errors were included. Flight duration was 150 s, satellite-orbit error was less than 10 m, pseudorange error was less than 10 m, satellite-speed error was 0.1 m/s, and height error was 1 m in a typical single-satellite environment. The INS adopted the same simulation environment as described above. Target motion speed was 20 m/s and direction was mainly in the *y* axis. Total simulation time was 2700 s. Due to the solution, only 2700 s of results were output. The results are shown in Figure 8 and Figure 9.

Our proposed single-satellite positioning algorithm based on UKF operated smoothly with no evident divergence under the comprehensive environment. Mean value was less than 50 m; positioning convergence was rapid and could be achieved within 10 s of starting output, error was less than 0.1 km and positioning error was less than 100 m. Figure 9 shows the comparison of absolute position error (reported as RMS) under the comprehensive environment for the three single-satellite positioning algorithms. It shows that the mean error between our proposed algorithm and the UKF algorithm was much smaller than that of the least-squares Doppler single-satellite algorithm. The mean error of our proposed algorithm was only 3.9% of the least-squares algorithm and the EKF algorithm was 8.6% of the least-squares algorithm.

### 4.5. Real-Environment Testing

**Figure 10:**
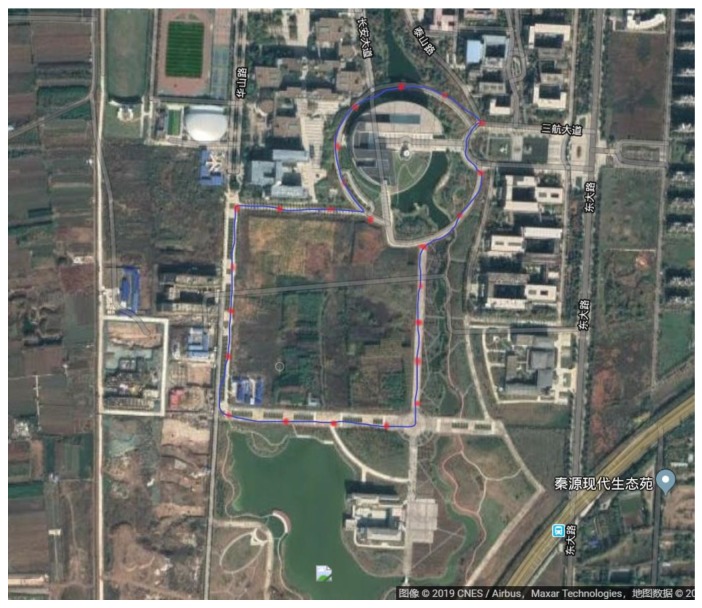
Real-environment testing map.

**Figure 11:**
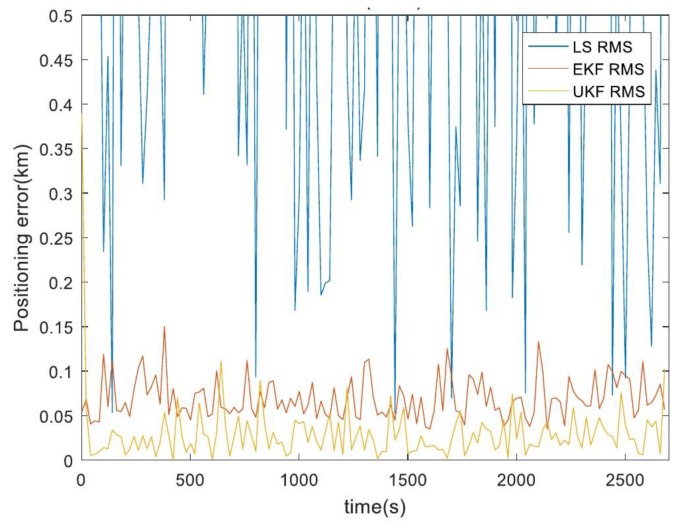
Real-environment testing of different algorithms.

In the real-environment test, we used a four-rotor unmanned aerial vehicle that was produced by the Dji company and a Hongyan satellite-enhancement system that provides a single-satellite navigation and positioning service to achieve real testing; testing time was 30 min.The test site was in the village of Dongda of the city of Xi’an which is shown in Figure 10. The blue line is the flight route of unmanned aerial vehicle, the red point is the ideal path passing point which obtained by GPS and wireless beacon, the real-environment test results are shown in Figure 11.

Figure 11 shows the real-environment testing results for the different algorithms. The average error of our proposed algorithm, obtained by 100 independent repeated calculations, was only 46.6% of that of the EKF algorithm and far superior to LS algorithm.The actual test results were close to the comprehensive simulation results. The test results show that our proposed single-satellite positioning algorithm based on UKF can effectively eliminate orbit, satellite-velocity and pseudorange errors and that it significantly improves the accuracy of single-satellite dynamic positioning.

## 5. Conclusions

This article outlined an inertial-navigation-aided single-satellite highly dynamic positioning algorithm. Aiming at the problem of divergence and poor positioning accuracy of existing single-satellite systems for dynamic targets, a Doppler pseudorange and altitude information were adopted. First, the least-squares method combined with UKF was utilized to achieve fast convergence and the initial filter parameters. Then, the UKF filter was directly utilized to obtain stable final positioning results in a smooth transition. In this paper, the single error and comprehensive error that have a great influence on single-satellite positioning were simulated and compared with the Doppler single-satellite positioning algorithm based on least-squares Newton iteration and the Doppler single-satellite positioning method combined with EKF. The simulation and testing results proved that our proposed algorithm’s position accuracy was much better than that of the other algorithms. Under GNSS system-rejection conditions, it has strong practical value.
